# Preparation of Alcohol Dehydrogenase–Zinc Phosphate Hybrid Nanoflowers through Biomimetic Mineralization and Its Application in the Inhibitor Screening

**DOI:** 10.3390/molecules28145429

**Published:** 2023-07-15

**Authors:** Mao-Ling Luo, Hua Chen, Guo-Ying Chen, Shengpeng Wang, Yitao Wang, Feng-Qing Yang

**Affiliations:** 1 School of Chemistry and Chemical Engineering, Chongqing University, Chongqing 401331, China; 20185486@cqu.edu.cn (M.-L.L.); chenhuacqu@cqu.edu.cn (H.C.); 20221801017@stu.cqu.edu.cn (G.-Y.C.); 2 State Key Laboratory of Quality Research in Chinese Medicine, Institute of Chinese Medical Sciences, University of Macau, Macao, China; ytwang@um.edu.mo

**Keywords:** alcohol dehydrogenase, organic-inorganic hybrid nanoflowers, *Penthorum chinense* Pursh, enzyme inhibitors

## Abstract

A biomimetic mineralization method was used in the facile and rapid preparation of nanoflowers for immobilizing alcohol dehydrogenase (ADH). The method mainly uses ADH as an organic component and zinc phosphate as an inorganic component to prepare flower-like ADH/Zn_3_(PO_4_)_2_ organic-inorganic hybrid nanoflowers (HNFs) with the high specific surface area through a self-assembly process. The synthesis conditions of the ADH HNFs were optimized and its morphology was characterized. Under the optimum enzymatic reaction conditions, the Michaelis-Menten constant (*K_m_*) of ADH HNFs (β-NAD^+^ as substrate) was measured to be 3.54 mM, and the half-maximal inhibitory concentration (IC_50_) of the positive control ranitidine (0.2–0.8 mM) was determined to be 0.49 mM. Subsequently, the inhibitory activity of natural medicine *Penthorum chinense* Pursh and nine small-molecule compounds on ADH was evaluated using ADH HNFs. The inhibition percentage of the aqueous extract of *P. chinense* is 57.9%. The vanillic acid, protocatechuic acid, gallic acid, and naringenin have obvious inhibitory effects on ADH, and their percentages of inhibition are 55.1%, 68.3%, 61.9%, and 75.5%, respectively. Moreover, molecular docking analysis was applied to explore the binding modes and sites of the four most active small-molecule compounds to ADH. The results of this study can broaden the application of immobilized enzymes through biomimetic mineralization, and provide a reference for the discovery of ADH inhibitors from natural products.

## 1. Introduction

Alcohol dehydrogenase (ADH), which is a key enzyme for the metabolism of short-chain alcohols in organisms, is abundant in human and animal liver, and plant and microbial cells [1,2,3,4]. As a substrate-specific zinc-containing metalloenzyme [5], each enzyme subunit in the ADH structure binds to two Zn^2+^ that maintain the structure and the catalytically active center of the enzyme. ADH plays an important role in many physiological processes of human body. ADH catalyzes the oxidation of primary and secondary alcohols to produce corresponding aldehydes and ketones [6], in which the cofactor nicotinamide adenine dinucleotide (NAD^+^) is reduced to NADH [7]. Studies have reported that in alcoholism or cirrhosis, ADH activity is significantly increased in serum [8,9]. The initial activity of ADH is positively correlated with liver damage-related indicators [10]. ADH inhibitors can reduce the production of aldehydes in alcohol metabolism by inhibiting the activity of ADH, thereby acting as the alcoholism antidote and hepatoprotector [11]. Currently, the mainly used antidotes in clinical practice are synthetic ADH inhibitors, such as 4-methylpyrazole, cimetidine, and formamides [7]. The 4-methylpyrazole can also prevent acetaminophen-induced acute kidney injury [12]. However, the 4-methylpyrazole is not indicated for patients with metabolic acidemia [13], and may cause several adverse reactions, including headache, nausea, and dizziness [14]. Several natural medicines possess specific anti-alcoholism and hepatoprotective effects, which have significant clinical curative efficacy with few side effects [15,16]. Thus, screening of ADH inhibitors from natural medicines becomes a new approach to developing alcoholism antidotes. 

*Penthorum chinense* Pursh (*P. chinense*, Saxifragaceae) is a traditional Chinese medicine, which is named ‘GanHuanCao’ in Chinese [17]. It has the effects of clearing heat and detoxification, reducing icteric and dampness, activating blood circulation, and dissolving stasis and swelling, being used in the treatment of jaundice, edema, trauma, cholecystitis, and liver disease [18]. Furthermore, several previous studies reported that the aqueous extract of *P. chinense* can protect against both acute and chronic alcohol-induced liver injury [19], and displays antioxidative and free radical scavenging activities [20]. Therefore, the screening of ADH inhibitors from *P. chinense* is significant for exploring its pharmacological activity such as the protective effect on alcohol-induced liver injury.

Ge et al. [21] first reported a method for preparing protein-inorganic hybrid nanoflowers with certain morphology and structure using protein as an organic component and copper phosphate (Cu_3_(PO_4_)_2_) as an inorganic component. It was found that the laccase and carbonic anhydrase nanoflowers have higher catalytic activity than their corresponding free enzymes. Different from the traditional enzyme immobilization method, this biomimetic mineralization method can be realized by simply adding organic biological macromolecules to the inorganic metal ion solution, forming organic-inorganic hybrid nanoflowers. Therefore, the application of protein-inorganic hybrid nanoflowers for the immobilization of enzymes has received dominant attention owing to its simple and efficient synthesis method [22]. In recent years, organic-inorganic hybrid nanoflowers using lipase [23], horseradish peroxidase [24], glucose oxidase [25], α-amylase [26], urease [27], trypsin [28], chymotrypsin [29], papain [30], α-glucosidase [31], β-galactosidase [32], sucrose phosphorylase [33], and DNA [34] as organic components have been reported, and their catalytic activity and stability are higher than those of their corresponding free enzymes.

**Figure 1:**
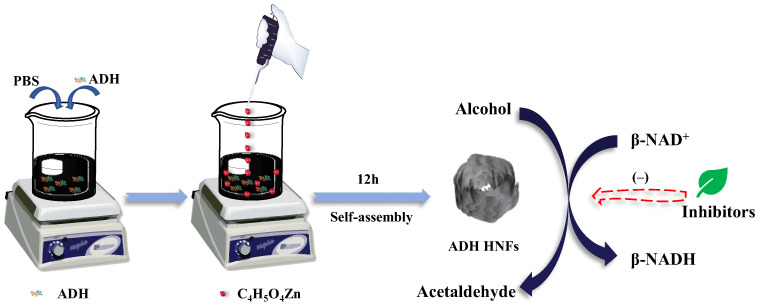
Schematic illustration of the fabrication process of ADH HNFs and the principle of catalysis.

In this study, ADH-zinc phosphate hybrid nanoflowers were prepared through biomimetic mineralization and applied in the screening of ADH inhibitors from *P. chinense* (Figure 1). The ADH hybrid nanoflowers (HNFs) were prepared using ADH as an organic component and Zn_3_(PO_4_)_2_ as an inorganic component, in which the synthesis of nanoflower carriers and the fixation of ADH were completed simultaneously, greatly simplifying the immobilization process of enzymes. The enzyme immobilization conditions were optimized, and the enzymatic reaction conditions and the kinetic performance of immobilized ADH were studied. Subsequently, the ADH inhibitory activity of aqueous extract and nine small-molecule compounds from *P. chinense* were evaluated using the prepared ADH HNFs. Finally, molecular docking of ADH with small-molecule compounds of good inhibitory activity was performed to predict the binding site of ligands to the enzyme.

## 2. Results and Discussion

### 2.1. Monitoring the Enzyme Activity of ADH HNFs by UV Analysis

**Figure 2:**
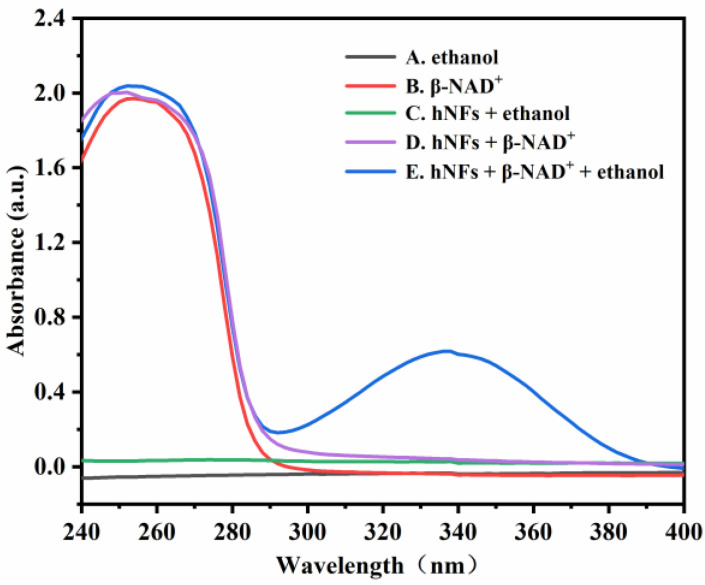
UV absorption spectra of five different solutions. (**A**) ethanol; (**B**) β-NAD^+^; (**C**) HNFs + ethanol; (**D**) HNFs + β-NAD^+^; (**E**) HNFs + β-NAD^+^ + ethanol. Conditions: the volumes of ADH HNFs, tris-HCl (pH = 7.0), and ethanol are 25, 200, and 50 μL, respectively; β-NAD^+^ final concentration, 3 mM; enzymatic reaction at 35 °C for 2 min and centrifuged for 1.5 min, 60 μL of supernatant was taken for measurement.

To investigate the feasibility of monitoring the enzyme activity by the design principle, the UV absorbances of five solutions at 240–400 nm were measured. As shown in Figure 2, both ethanol and HNFs + ethanol solutions (Figure 2A,C) have no UV absorption peaks at 240–400 nm. In the conversion of ethanol to acetaldehyde catalyzed by ADH, coenzyme I (β-NAD^+^) is involved to generate β-NADH. Both the β-NAD^+^ and β-NADH have absorption peaks at 260 nm (Figure 2B,D,E), but only the β-NADH shows a characteristic absorption peak at 340 nm (Figure 2E). Therefore, the enzymatic activity of ADH HNFs can be assessed by monitoring the absorbance of β-NADH at 340 nm. 

### 2.2. Optimization of Preparation Conditions of ADH HNFs

**Figure 3:**
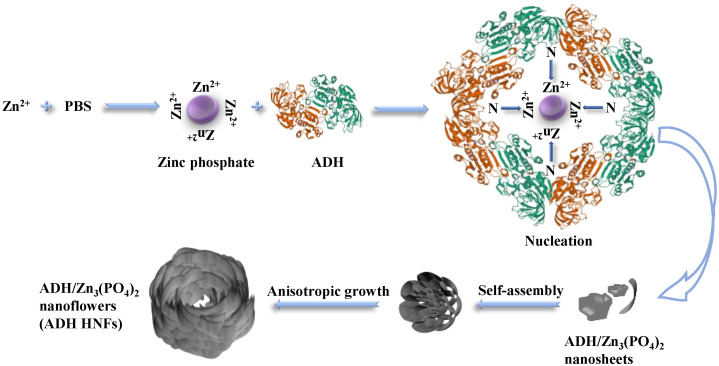
Schematic diagram of the proposed mechanism for the formation of ADH HNFs through the biomineralization method.

For the growth of hybrid nanoflower, there are four main steps, including crystallization and coordination, in situ precipitation, self-assembly, and size growth (Figure 3) [35]. The primary crystals of zinc phosphate are first formed. The complexes of enzyme molecules with Zn^2+^ are formed predominantly through the coordination facility of amide groups in the ethanol dehydrogenase backbone and nitrogen atoms in amino acid residues. These complexes in turn provide nucleation sites for the primary crystals. Next, the growth of Zn_3_(PO_4_)_2_ crystals at the Zn^2+^ binding site on the surface of the complex allows the ethanol dehydrogenase molecule and the primary crystals to gradually grow into large agglomerates, resulting in the formation of individual nanosheets. Then, the self-assembly occurs between nanosheets to form a flower-like structure, and finally, anisotropic growth results in the complete formation of ADH HNFs. In this proposed growth process, alcohol dehydrogenase plays an important role in regulating the nucleation of Zn_3_(PO_4_)_2_ crystals to form the scaffold for the petals and serves as a ‘glue’ to bind the nanosheets together [21].

To obtain ADH HNFs with suitable size and morphology, as well as good catalytic activity and storage stability, the preparation method, enzyme amount, and immobilization time were investigated. The magnetic stirring equipment used in this experiment cannot be temperature controlled and the room temperature was about 25 °C during the synthesis, the effect of different temperatures on the preparation of nanoflowers is not considered here. Furthermore, according to the previous reports [36,37,38], phosphate organic-inorganic hybrid nanoflowers are usually fully formed within 6 h, therefore, 6 h was used firstly as the time to immobilize ADH. The reproducibility of the immobilized enzyme can be used to appraise whether the immobilization method is stable and reliable. Three batches of ADH HNFs were prepared through different preparation methods (thermostatic oscillation for 6 h at 30 °C or magnetic stirring for 6 h at room temperature of 30 °C) and evaluated by measuring the absorbance of generated β-NADH at 340 nm. The relative standard deviation (RSD) of batch-to-batch (*n* = 3) for magnetic stirring and thermostatic oscillation is 4.1% and 9.4%, respectively, indicating that the ADH HNFs prepared by magnetic stirring have better reproducibility.

**Figure 4:**
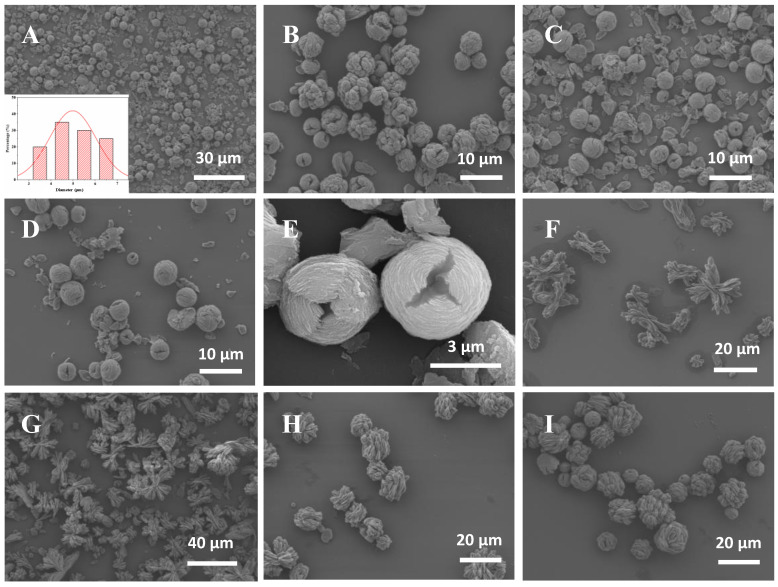
The SEM images of ADH HNFs that were prepared through magnetic stirring for 6 h at room temperature (**A**–**E**) and thermostatically oscillation for 6 h at 30 °C (**F**–**I**). The enzyme amounts of (**A**,**B**,**C**,**D**,**E**,**F**,**G**,**H**,**I**) are 6, 4, 6, 8, 6, 2, 4, 6, and 8 mg, respectively. The insert in Figure (**A**) shows the particle size distribution of the nanoflower prepared with 6 mg of enzyme amount.

As shown in Figure 4A–I, the hybrid nanoflowers prepared through different methods have obviously different morphologies and sizes. Figure 4A–E shows that ADH HNFs prepared through the magnetic stirring method have a tight structure, approximating the structure of a ‘flower bud’. Figure 4F–I shows that ADH HNFs prepared through the constant temperature oscillation method have sparse petals and loose structures. The overall size is larger than that of ADH HNFs prepared through the magnetic stirring method. Based on these SEM images, the poor reproducibility of the ADH HNFs prepared by the thermostatic oscillation method may be due to the non-uniform flower structure of the nanoflowers prepared in different batches.

The SEM images of ADH HNFs synthesized through magnetic stirring under the optimum conditions (6 mg of enzyme amount and 12 h of immobilization time) are shown in Figure 4A,C,E. The nanoflowers are uniformly dispersed with similar particle sizes within the field of view, and no large number of agglomerates are found. Furthermore, it can be seen in Figure 4A that in addition to the structures bound as nanoflowers, there are also some incompletely assembled nanosheets, which confirms that the formation of nanoflowers is a self-assembly and gradual growth process. From the SEM image shown in Figure 4E and particle size distribution diagram (Figure 4A), the nanoflowers are spheres in shape with a diameter size of 4–6 µm, and the petals are arranged in a wrapped shape.

**Figure 5:**
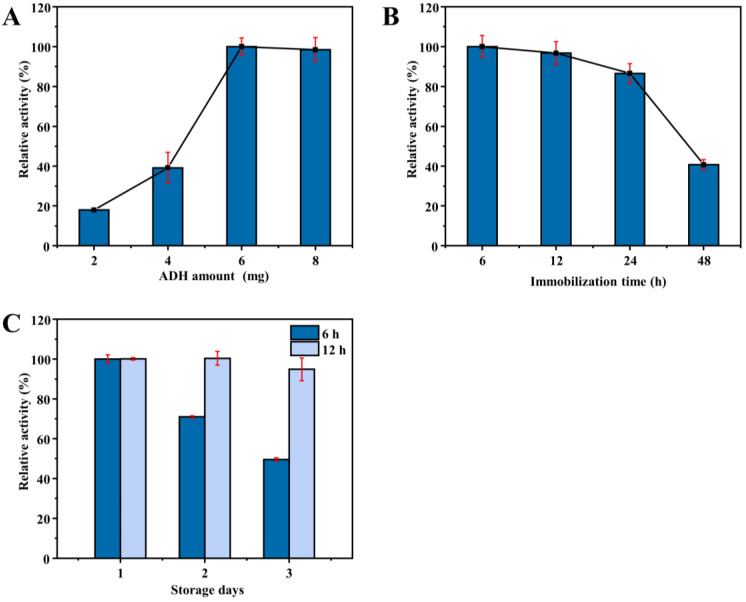
Effect of enzyme amount (**A**) and immobilization time (**B**) on the catalytic activity of ADH HNFs prepared through magnetic stirring; effect of immobilization time (6 and 12 h) on the storage stability of ADH HNFs (**C**). Conditions: the volume of ADH HNFs, tris-HCl (pH 7.0), and ethanol are 15, 160, and 50 µL, respectively; β-NAD^+^ concentration, 3 mM; reaction temperature, 35 °C; reaction time, 2 min; centrifuged for 1.5 min, 60 μL of supernatant was taken for measurement at 340 nm.

Then, the effect of enzyme amount on the catalytic activity of ADH HNFs prepared through magnetic stirring was investigated. The relative catalytic activity of ADH HNFs increased significantly in the range of 2–6 mg of the enzyme amount and decreased slightly to 8 mg (Figure 5A). In consideration of the cost-saving and good catalytic activity, the enzyme amount of 6 mg was chosen for the following studies. Furthermore, the relative catalytic activity of ADH HNFs is gradually decreased with the increase of immobilization time (Figure 5B), but the storage stability of the nanoflowers immobilized for 12 h is better than 6 h (Figure 5C). Therefore, 12 h was chosen as the optimum immobilization time. In addition, the relationship between immobilization time and relative enzyme activity confirms that it is reasonable to choose 6 h as the synthesis time when comparing different immobilization methods.

### 2.3. Optimization of Enzymatic Reaction Conditions

To obtain the optimum reaction conditions for ADH HNFs, several experimental parameters were investigated, including reaction temperature (25, 35, and 45 °C), buffer pH (6.5, 7.0, and 7.5), and reaction time (2.0–10.0 min).

**Figure 6:**
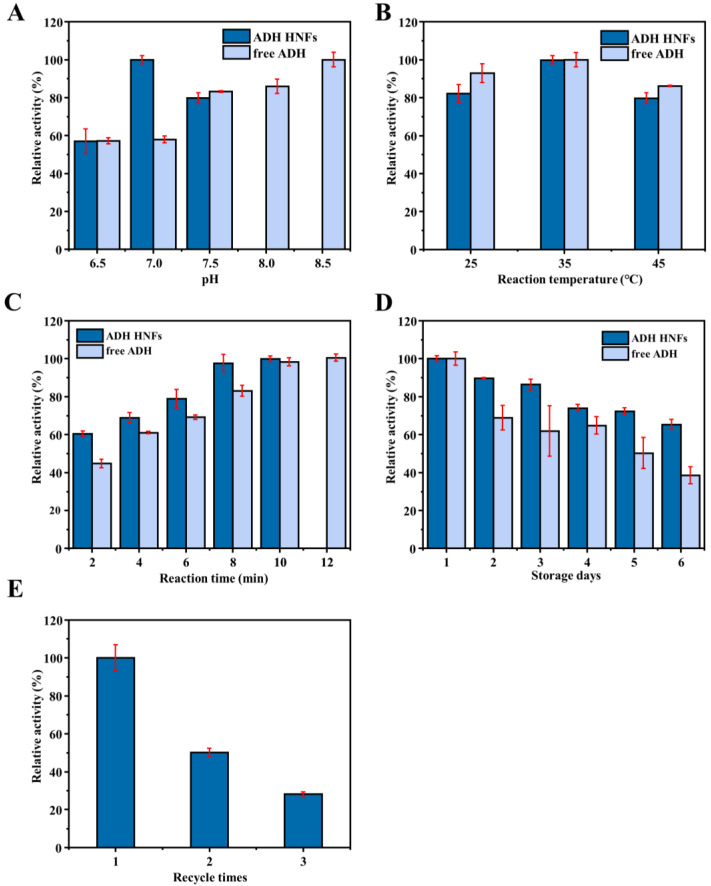
Effect of buffer pH (**A**), reaction temperature (**B**), reaction time (**C**), and the storage stability (**D**) on the relative catalytic activity of ADH HNFs and free ADH, and reusability (**E**) of ADH HNFs. ADH HNFs conditions: the volume of ADH HNFs, tris-HCl, and ethanol are 15, 160, and 50 µL, respectively; β-NAD^+^ concentration, 3 mM; buffer pH, 7.0; reaction temperature, 35 °C; reaction time, 2 min for (**A**,**B**), 8 min for (**D**,**E**); centrifuged for 1.5 min, 60 μL of supernatant was taken for measurement at 340 nm. Free ADH conditions: the volume of tris-HCl and ethanol are 160 µL and 15 µL, respectively; free ADH concentration, 0.015 mg/mL for (**A**–**C**), 0.01 mg/mL for (**D**); β-NAD^+^ concentration, 3 mM; buffer pH, 7.5; reaction temperature, 35 °C; reaction time, 2 min for (**A**,**B**), 8 min for (**D**); 60 μL of supernatant was taken for measurement at 340 nm.

Results indicate that the relative catalytic activity of ADH HNFs reached its maximum at pH 7.0, while free ADH was leveling off at pH 7.5 (Figure 6A). At the same time, as the reaction temperature increased from 25 to 45 °C, the catalytic activity of both free ADH and ADH HNFs peaked at 35 °C (Figure 6B), which was chosen as the optimum temperature. Moreover, with the increase in reaction time (2, 4, 6, 8, 10, and 12 min), the catalytic activity of ADH HNFs is increased and reaches its maximum at 8 min, and free ADH reaches its maximum at 10 min (Figure 6C). Thus, 8 min and 10 min of reaction time for ADH HNFs and free ADH were used in the following experiments, respectively.

The storage stability and reusability of ADH HNFs were also evaluated and the results are shown in Figure 6D,E, respectively. Within six days of storage, the catalytic activity of the free enzyme decreased to 45.1%, but ADH HNFs remained at 65.3% (Figure 6D). Furthermore, as shown in Figure 6E, ADH HNFs can retain a certain level of catalytic activity after three times of usage. These results indicate that the immobilization of ADH through the formation of nanoflowers can improve the operational stability of the enzyme. 

### 2.4. Kinetics Study of ADH HNFs

**Figure 7:**
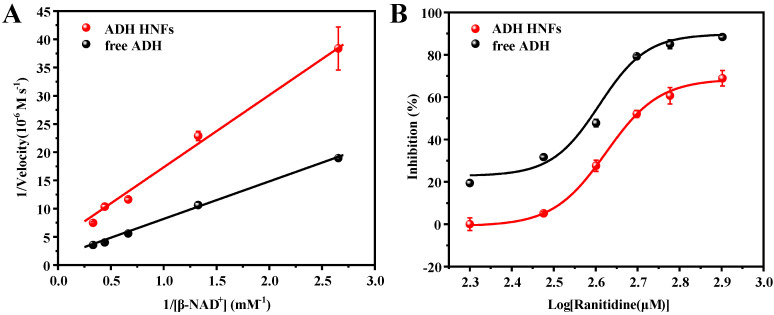
Michaelis–Menten double reciprocal curve (**A**) and inhibition plot of ranitidine on ADH (**B**). ADH HNFs conditions: the volume of ADH HNFs, tris-HCl, and ethanol are 15, 160, and 50 µL, respectively; β-NAD^+^ concentration, 3 mM; buffer pH, 7.0; reaction temperature, 35 °C; reaction time, 8 min. β-NAD^+^ concentrations are from 0.375 to 3 mM for (**A**). Ranitidine concentrations are from 0.2 to 0.8 mM for (**B**); centrifuged for 1.5 min, 60 μL of supernatant was taken for measurement at 340 nm. Free ADH conditions: The volume of tris-HCl and ethanol are 160 and 50 µL, respectively; free ADH concentration, 0.015 mg/mL; buffer pH, 7.5; reaction temperature, 35 °C; reaction time, 10 min, 60 μL of supernatant was taken for measurement at 340 nm.

The steady-state kinetic study of ADH HNFs was carried out. Based on the varied β-NAD^+^ concentrations (0.38, 0.75, 1.51, 2.26, and 3.0 mM), the linear regression equations of the Lineweaver–Burk plot of ADH HNFs and free ADH are obtained through Equation (1) as y = 13.231x + 3.8107 (R^2^ = 0.993) and y = 6.729x + 1.2614 (R^2^ = 0.9981), respectively, where y and x are the reaction velocity and reciprocal of substrate (β-NAD^+^) concentration, respectively (Figure 7A). The *V_max_* of ADH HNFs and free ADH are 15.72 and 47.58 μM·min^−1^, and *K_m_* values are 3.47 and 5.33 mM, respectively, in which the *K_m_* value of ADH HNFs is comparable to that of free ADH. In addition, as can be seen in Table 1, the *K_m_* values obtained from different studies are different, which can be attributed to the source of ADH, the concentration of the substrate, and the conditions of the enzymatic reaction. Moreover, the *K_cat_* value of free ADH (0.9366 min^−1^) was found to be a little higher than that of ADH HNFs (0.4546 min^−1^). Because *K_cat_* demonstrates the maximum converted substrate molecule number by an enzyme per unit of time, a decrease in *K_cat_* value reflects the reduced activity of the enzyme [39].

The change in the amounts of product produced with different ranitidine concentrations was measured and the inhibition rate at each concentration was calculated through Equation (2). Then, the inhibition plots of ranitidine against ADH HNFs were obtained through the dose–response nonlinear regression equation using Origin 2018 64 c software (Figure 7B). The IC_50_ value of positive control ranitidine against ADH HNFs and free ADH are calculated to be 0.49 and 0.39 mM according to the fitted equation.

### 2.5. Screening of Inhibitors and Molecular Docking

The inhibitory activity of the aqueous extract of *P. chinense* (8 mg/mL) and nine pure compounds (1.5 mM) on ADH HNFs were evaluated. Compared with the blank group without inhibitor, the inhibitory activity of each sample was calculated through Equation (2), and the results are summarized in Table 2. The inhibition percentage of the aqueous extract is 57.9%, indicating that *P. chinense* possesses a moderate inhibitory effect on ADH. The process of ADH alcohol metabolism is associated with increased oxidative stress, which may lead to liver inflammation and cellular damage [46]. The aqueous extract of *P. chinense* can inhibit the activity of ADH and reduce the production of aldehydes in alcohol metabolism, thereby playing the role of alcoholism antidote and hepatoprotector. Among the nine small-molecule compounds from *P. chinense*, vanillic acid, protocatechuic acid, gallic acid, and naringenin show obvious inhibitory effects on ADH with the % of inhibition of 55.1%, 68.3%, 61.9%, and 75.5%, respectively. Therefore, these active compounds may be responsible for the inhibitory activity of *P. chinense* on ADH, and the immobilized enzyme (ADH HNFs) has the potential application prospect in inhibitor screening.

**Figure 8:**
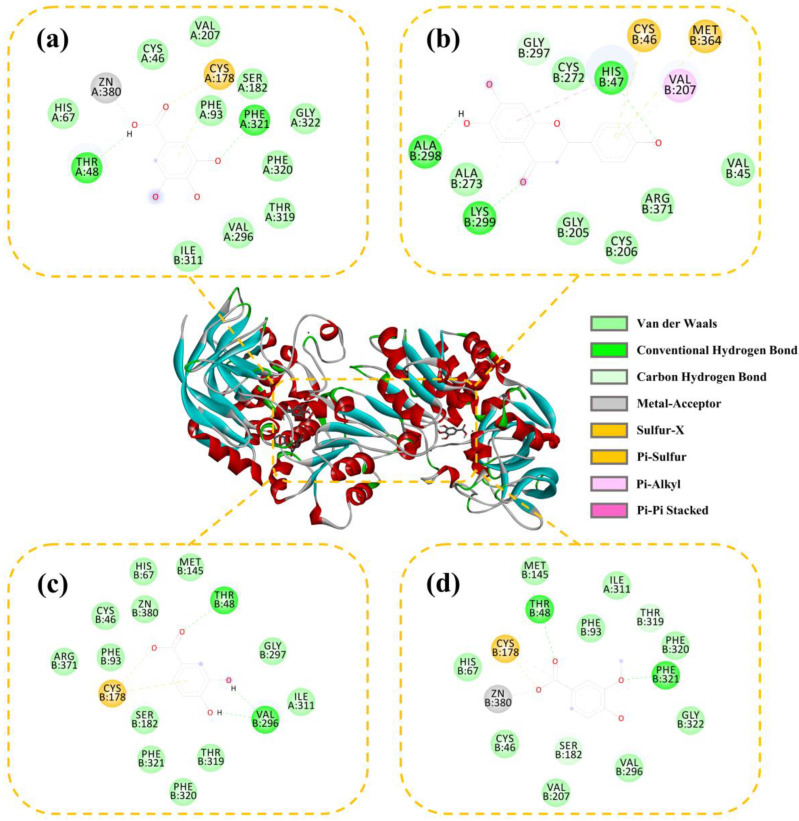
Three-dimensional (3D) and 2D docking images of ADH with gallic acid (**a**), naringenin (**b**), protocatechuic acid (**c**), and vanillic acid (**d**).

**Figure 9:**
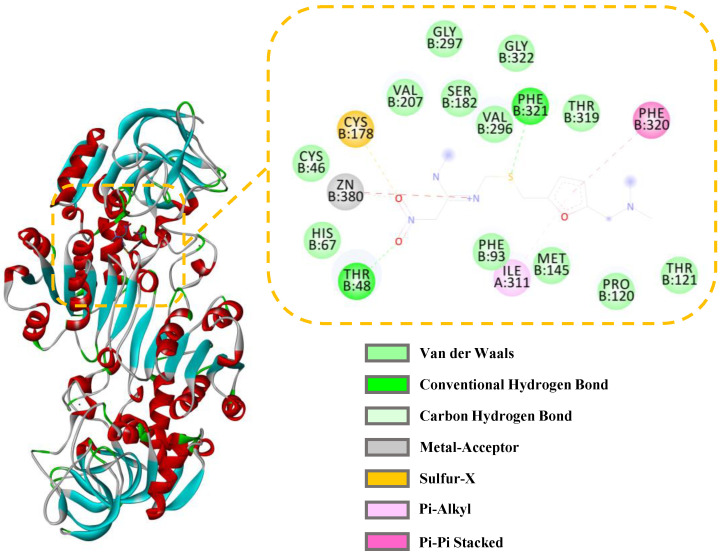
Three-dimensional (3D) and 2D docking images of ADH with ranitidine.

The interactions between ADH and the active compounds (potential inhibitors) were investigated through molecular docking, including ranitidine, vanillic acid, protocatechuic acid, naringenin, and gallic acid. Figure 8 and Figure 9 display the interaction diagrams of the best-docked conformations. Table 3 summarizes the amino acid residues, hydrogen bonds, and binding energy of the interactions between the small-molecule compounds and ADH. The molecular docking results show that ranitidine, vanillic acid, protocatechuic acid, naringenin, and gallic acid are all located in the active site cavity of ADH, and their binding energy to the docking region of the enzyme is below −5 Kcal/mol, indicating that these four small molecules may be potential ADH inhibitors [47]. Furthermore, hydrogen bonds that exist between vanillic acid, gallic acid, and PHE321, THR48; protocatechuic acid and VAL296, THR48; naringenin and HIS47, ALA298, LYS299; play important roles in the binding of ligands to ADH. Compared with the positive control ranitidine, vanillic acid, gallic acid, and protocatechuic acid have some similar binding amino acid residues. Particularly, the benzene rings of these potential inhibitors make close contacts with CYS46, CYS178, HIS67, ILE311, THR319, PHE93, PHE320, ZN380, and SER182, which are the residues lining the inner and middle part of the substrate-binding pocket together with the catalytic zinc and the zinc ligands [48]. However, naringenin exhibits a different binding site from the other three small-molecule compounds and mainly interacts with HIS47, ALA298, and LYS299, which can be used to explain its higher inhibitory activity than the other three ones. Therefore, these compounds may be effective ADH inhibitors, and the docking results correspond to that of inhibitor screening.

## 3. Materials and Methods

### 3.1. Chemicals and Materials

Alcohol dehydrogenase (ADH, 300 U/mg, from yeast) was obtained from Shanghai Yingxin Laboratory Equipment Co., Ltd. (Shanghai, China). Zinc acetate dihydrate (C_4_H_5_O_4_Zn·2H_2_O) and sodium phosphate dibasic dodecahydrate (Na_2_HPO_4_·12H_2_O) were purchased from Shanghai Titan Scientific Co., Ltd. (Shanghai, China). Oxidative coenzyme I (β-NAD, ≥98%) and ranitidine hydrochloride (≥98%) were obtained from Shanghai Yuanye Biotechnology Co., Ltd. (Shanghai, China). Potassium dihydrogen phosphate (KH_2_PO_4_), potassium chloride (KCl), and sodium chloride (NaCl) were purchased from Chengdu Chron Chemicals Co., Ltd. (Chengdu, China). Hydrochloric acid (HCl) was purchased from Sichuan Xilong Science Co., Ltd. (Chengdu, China). Tris (hydroxymethyl) aminomethane (C_4_H_11_NO_3_) was obtained from Guangdong Guanghua Sci-Tech Co., Ltd. (Shantou, China). Vanillic acid (≥98%), (+)-catechin hydrate (>98%), protocatechuic acid (≥97%), ellagic acid (≥96%), and brilliant blue G were obtained from Shanghai Aladdin Biochemical Technology Co., Ltd. (Shanghai, China). Naringenin (>98%), gallic acid (≥98%), apigenin (>98%), and epicatechin (>98%) were purchased from Chengdu PureChem-Standard Co., Ltd. (Chengdu, China). Quercetin (≥98%) was obtained from Sinopharm Chemical Reagent Co., Ltd. (Shanghai, China). *Penthorum chinense* Pursh was obtained from Anguo Guangsheng Trading Co., Ltd. (Anguo, China).

### 3.2. Instruments

A DF-101S collector constant temperature magnetic stirrer (Zheng Great Wall Science Industry and Trade Co., Ltd., Zhengzhou, China) and a DHG-9146A electric heating constant temperature blast drying shaker (Shanghai Longyue Instrument Equipment Co., Ltd., Shanghai, China) were used for the synthesis of the materials. The SEM images for the characterization of material were obtained through a field-emission SEM (JSM-7600F, JEOL Ltd., Tokyo, Japan). The pH of solutions was measured through a FE 28 pH meter (Mettler-Toledo Instruments, Shanghai, China). A UV-5500 PC spectrophotometer (Shanghai Metash Instruments Co., Ltd., Shanghai, China) was used for the UV-Vis analysis. The tabletop low-speed centrifuge L420 and the ultrasonic cleaner used in this study were obtained from Hunan Xiang Yi Laboratory Instrument Development Co., Ltd. (Changsha, China) and Kunshan Jielimei Ultrasonic Instrument Co., Ltd. (Kunshan, China), respectively. The ultrapure water prepared through a water purification system (ATSelem 1820A, Antesheng Environmental Protection Equipment, Chongqing, China) was used for all experiments.

### 3.3. Synthesis of ADH HNFs 

Phosphate (PBS, pH = 7.13) buffer for ADH HNFs was prepared by dissolving 0.56 g Na_2_HPO_4_·12H_2_O, 0.14 g KH_2_PO_4_, 0.098 g NaCl, and 0.017 g KCl in ultrapure water, respectively, and fixing the volume to 100 mL [49]. To prepare ADH/Zn_3_(PO_4_)_2_ hybrid nanoflowers, 6 mg of ADH was dissolved in 5 mL of PBS buffer. Afterward, 0.4 mL of zinc acetate solution was slowly added to the buffer and stirred magnetically for 12 h (Figure 1). The suspension was then centrifuged at 3000 rpm for 5 min and the supernatant was removed and the precipitate was washed twice with PBS buffer to remove the unfixed enzyme and the unreacted zinc acetate. Finally, the prepared ADH HNFs were suspended in 4 mL of PBS buffer and stored in the refrigerator at 4 °C.

### 3.4. Optimization of Preparation Conditions of ADH HNFs

Different enzyme amounts of 2, 4, 6, and 8 mg were introduced and reacted under constant temperature shaking (30 °C, 160 rpm) or magnetic stirring (room temperature of 30 °C) for 6 h, respectively. The unit of alcohol dehydrogenase activity is defined as the amount of 1 μmol NADH·min^−1^ consumed by alcohol dehydrogenase under specified conditions [50]. Subsequently, field emission scanning electron microscopy (SEM) was used to observe the morphology and size of the ADH HNFs synthesized by using different enzyme amounts and preparation methods. Finally, the enzyme concentration was determined using the Bradford method [51].

### 3.5. Determination of ADH HNFs Enzymatic Activity

The conversion of coenzyme I (β-NAD^+^) to β-NADH during the catalysis of ethanol to acetaldehyde by ADH displays a characteristic absorption peak at 340 nm. In a 0.5 mL centrifuge tube, 15 μL of ADH HNFs (dispersed in PBS, pH = 7.13), 160 μL of tris-HCl (prepared in 10 mM tris solution and the required pH was adjusted by 1 M HCl), 50 μL of anhydrous ethanol, and 25 μL of 3 mM of β-NAD^+^ (dispersed in tris-HCl, pH = 7.0) were mixed well, and the mixture was incubated at 35 °C for 8 min. After being centrifuged for 1.5 min by a portable mini centrifuge, the absorbance changes at 340 nm (ε = 6200 M^−1^ cm^−1^ [41]) of 60 μL of supernatant was measured. Each experiment was repeated three times.

### 3.6. Determination of Free ADH Enzymatic Activity

In a 0.5 mL centrifuge tube, 15 μL of free ADH (0.25 mM, dispersed in PBS, pH = 7.13), 160 μL of tris-HCl (prepared in 10 mM tris solution and the required pH was adjusted by 1 M HCl), 50 μL of anhydrous ethanol, and 25 μL of 3 mM of β-NAD^+^ (dispersed in tris-HCl, pH = 7.5) were mixed well, and the mixture was incubated at 35 °C for 10 min. The absorbance (340 nm) of 60 μL of supernatant was measured. Each experiment was repeated three times.

### 3.7. Enzyme Kinetics Assay

For an enzyme kinetic reaction, the *K_m_* (Michaelis–Menten constant) is an important parameter that can reflect the affinity between the substrate and enzyme, which is calculated through the Lineweaver–Burk Equation (1) [52].

(1) $$\frac{1}{V}=\frac{K_{m}}{V_{max}\left[S\right]}+\frac{1}{V_{max}}$$

where [S] is the concentration of the substrate (β-NAD^+^), *V_max_* and *V* are the maximum and reaction rate of the enzyme reaction, respectively. The reaction velocity of ADH HNFs was monitored through the absorbance (340 nm) of the product (β-NADH). Experiments were performed by altering the concentration of β-NAD^+^ under optimized conditions.

Ranitidine is a representative inhibitor of ADH [53], which was used as a model compound for evaluating the inhibition kinetics of ADH. IC_50_ was measured by varying the concentration of ranitidine (0.2–0.8 mM) with a fixed β-NAD^+^ concentration at 3 mM. The % of inhibition (*I*%) can be calculated through Equation (2) [52]:

(2) $$I\left(\%\right)=\left(1-\frac{A_{i}}{A_{0}}\right)\times 100\%$$

where *A_i_* and *A*_0_ are the absorbances of the reaction product with and without the inhibitor, respectively. *I*(%) is the inhibitory percentage. The dose–response nonlinear regression equation was used for the construction of an inhibition plot using Origin 2018.

### 3.8. Evaluation of ADH Inhibitory Activity of P. chinense and Its Small-Molecule Compounds

The preparation of an aqueous extract of *P. chinense* was referred to as the previously reported method [54]. In brief, 10.0 g of crushed *P. chinense* was soaked in 150 mL of ultrapure water in a 250 mL round-bottomed flask for 2 h, and condensed and refluxed at 100 °C for 1 h, then it was filtered. After that, 100 mL of ultrapure water was added to the residue, which was condensed and refluxed at 100 °C for 40 min, and it was filtered. After combining the two filtrates, a solution equivalent to 0.04 g/mL of the original drug was prepared. 

Vanillic acid, (+)-catechin hydrate, gallic, epicatechin, and protocatechuic acid solutions were prepared by directly adding purified water to dissolve the corresponding substance. The ellagic acid, apigenin, and quercetin solutions were prepared by dissolving them in a small volume of lye (NaOH, 1 M) in ultrapure water. The naringenin solution was prepared in ethanol. The final concentrations of all solutions are 1.5 M.

The prepared ADH HNFs were used in the evaluation of the inhibitory activity of the aqueous extract and the monomers of *P. chinense*. In brief, 15 μL of ADH HNFs suspension was added to a 0.5 mL centrifuge tube, followed by 50 μL of the aqueous extract of *P. chinense* or 50 μL of the monomers, 110 μL of tris-HCl, and 50 μL of anhydrous ethanol, which were mixed with 25 μL of 3 mM of β-NAD^+^ and reacted at 35 °C for 8 min. Then, the reaction mixture was centrifuged for 1.5 min, and the absorbance of the product β-NADH at 340 nm was recorded in 60 μL of supernatant. The inhibition percentage of each sample was calculated according to Equation (2) based on the absorbance of enzymatic reaction product β-NADH with and without the addition of an inhibitor. At least three parallel experiments were carried out for each sample.

### 3.9. Molecular Docking

For the molecular docking analysis, the crystal structure of ADH (PDB ID: 1E3L) was obtained from the Protein Data Bank. Before molecular docking, the NAD ligand and H_2_O molecules in the ADH structure were removed in AutoDock 1.5.6, followed by the addition of polar hydrogen, calculation of point charge, and selection of atom type. Then, the processed ADH was output in PDBQT format. On the other hand, the molecular structures of the small-molecule compound were drawn in ChemDraw 20.0 and imported into Chem3D for energy minimization, and the results were saved in PDB format. Subsequently, the docking site and range of the enzyme were selected, and the processed ligands were docked with ADH using the Lamarckian genetic algorithm (LGA). Finally, the optimum conformations of ligands and ADH interactions were observed by Discovery Studio 2019.

## 4. Conclusions

In this study, ADH/Zn_3_(PO_4_)_2_ hybrid nanoflowers were successfully prepared through a simple biomimetic mineralization method. The SEM characterizations demonstrate the successful synthesis of ADH HNFs, and the kinetics of the enzymatic reaction of the nanoflowers shows that the immobilization method can effectively improve the affinity of the enzyme to the substrate. Furthermore, the catalytic activity of the immobilized enzyme shows better storage stability (6 days) and reusability (3 times) as compared to that of the free enzyme. In addition, the good inhibitory activity on ADH of nine small-molecule compounds from *P. chinense* has been discovered, and the molecular docking further confirmed the interaction between the enzyme and the four most active compounds, namely vanillic acid, protocatechuic acid, gallic acid, and naringenin. In short, the results of the present study provide references for the immobilization of enzymes through simple methods and the discovery of ADH inhibitors from natural products.

## Figures and Tables

**Table 1:** Comparison of present work with the previously reported methods for immobilization of ADH.

Immobilization Carrier	Substrate		*K_m_* (mM)		*V_max_* (μM·min^−1^)		*K_cat_* (min^−1^)		Ref.
			Immobilized	Free	Immobilized	Free	Immobilized	Free	
Fe_3_O_4_@SiO_2_-epoxy NPs	Ethanol	NAD^+^	31.32	11.54	44.27	56.72	– ^a^	–	[40]
HKUST-1	Ethanol	NAD^+^	34.3	26.2	2300	10700	13.5	62.6	[41]
Metal-chelated cryogels	Phenylglyoxylic acid	NAD^+^	35	143	0.034	71.43	3165.7	3743.9	[39]
Ni-Co nanoferrites	Ethanol	NAD^+^	237	154	190.83	315.55	–	–	[42]
Polyaniline coated AgNPs	Ethanol	NAD^+^	205.3	163.7	233.0	321.2	–	–	[43]
PPy-TiP	Ethanol	NAD^+^	223.71	153.6	201.53	340.7	–	–	[44]
TiO_2_ NPs	Formaldehyde	NAD^+^	23.3	11.5	65.8	100	1.45	2.2	[45]
HNFs	Ethanol	NAD^+^	3.54	5.33	15.72	47.58	0.4546	0.9366	This work

HKUST-1: a copper-containing metal framework organic material; NPs: nanoparticles; AgNPs: silver nanoparticles; PPy-TiP: polypyrrole–titanium(iv) phosphate nanocomposite; HNFs: hybrid nanoflowers; ^a^ it was not reported in the reference.

**Table 2:** The percentage of inhibition of nine compounds and *P. chinense* aqueous extracts on ADH (*n* = 3).

Compounds	Inhibition (%)	Compounds	Inhibition (%)
Aqueous extract of *P. chinense*	57.9 ± 1.2	L-Epicatechin	– ^a^
Apigenin	52.5 ± 5.5	Naringenin	75.5 ± 2.1
Cianidanol	34.9 ± 4.0	Protocatechuic acid	68.3 ± 2.9
Ellagic acid	58.2 ± 10.3	Quercetin	–
Gallic acid	61.9 ± 2.8	Vanillic acid	55.1 ± 1.0

*P. chinense*: *Penthorum chinense* Pursh; ^a^ no inhibition or inhibition rate less than 0.6%.

**Table 3:** Docking results of ranitidine and four small-molecule compounds with ADH.

Compounds	Binding Energy (Kcal/mol)	Hydrogen Bonds	Distance (10^−10^ m)	Other Amino Acid Residues
Ranitidine ^a^	−6.77	PHE321, THR48	2.00 2.65	CYS46, CYS178, GLY297, GLY322, HIS67, ILE311, MET145, VAL296, THR319, THR121, PRO120, PHE93, PHE320, ZN380, VAL207, SER182
Gallic acid	−7.94	PHE321, THR48	2.09 2.21	VAL207, CYS178, PHE93, SER182, GLY322, PHE320, THR319, VAL296, ILE311, HIS67, ZN380, CYS46
Naringenin	−5.53	HIS47, ALA298, LYS299	2.18 2.66 2.60	GLY297, CYS272, CYS46, MET364, VAL207, VAL45, ARG371, CYS205, CYS206, ALA273
Protocatechuic acid	−7.86	VAL296, THR48	1.93 2.38	MET145, GLY297, ILE311, THR319, PHE320, PHE321, SER182, CYS178, PHE93, ARG371, CYS46, ZN380, HIS67
Vanillic acid	−8.27	PHE321, THR48	1.79 2.48	MET145, PHE93, ILE311, THR319, PHE320, GLY322, VAL296, SER182, VAL207, CYS46, ZN480, HIS67, CYS178

^a^ ranitidine was used as a positive control and the molecular docking results are shown in Figure 9.

## Data Availability

The data presented in this study are contained within the article.

## Sample Availability

Samples of the compounds are available from the authors.
